# School bonding and ethos in trajectories of offending: Results from the Belfast Youth Development Study

**DOI:** 10.1111/bjep.12303

**Published:** 2020-02-17

**Authors:** Kathryn Higgins, Oliver Perra, Julie‐Ann Jordan, Tara O'Neill, Mark McCann

**Affiliations:** ^1^ Centre for Evidence and Social Innovation Queen's University Belfast UK; ^2^ School of Nursing and Midwifery Queen's University Belfast UK; ^3^ MRC/CSO Social and Public Health Sciences Unit University of Glasgow UK

**Keywords:** school, longitudinal, school commitment, school attachment, offending, multilevel modelling

## Abstract

**Background:**

Aspects of the school environment, such as school attachment levels, are linked to adolescent offending. Previous research has not clarified whether a school‐ or individual‐level intervention approach to improving pupil school attachment and commitment is most likely to reduce adolescent offending.

**Aim:**

The present study assessed the impact of individual‐ and school‐level variables on offending behaviour from ages 14–16 years.

**Sample:**

The participants were 4,049 young people from 42 mainstream schools who took part in the Belfast Youth Development Study.

**Method:**

Multilevel modelling was used to examine the relative influence of individual‐ and school‐level variables on offending behaviour in adolescence.

**Results:**

Pupils who had high levels of school commitment and attachment and were involved in fewer fights at age 13 reported lower levels of offending at age 14 years. Differences between schools accounted for 7% of the variation in offending. Lower individual‐level commitment was associated with higher initial levels of offending at age 14 if the school‐level ethos was of higher commitment. Lack of safety at the school level appeared to be detrimental for young people not exposed to socio‐economic deprivation.

**Conclusions:**

Individual‐level targeted interventions are likely to be a more cost‐effective approach of reducing offending behaviour in adolescence. Additional, albeit smaller, reductions in offending levels could be achieved through school‐level interventions in some school types (e.g., deprived areas).

## Background

### Youth offending and educational outcomes

Many youths start offending early in adolescence while they are still in school. Indeed, in 2012/13, 11–12% of arrests made in England, Wales, and Northern Ireland involved young people aged 10–17 years (Ministry of Justice, 2015; Police Service of Northern Ireland, 2015), and in the United States in the same year, 4% of youths were arrested for an offence (US Department of Justice, 2014). Furthermore, surveys measuring reported crime consistently show that recorded figures drastically underestimate offending, and therefore, the true level of youth offending is likely to be much higher (Office for National Statistics, 2015). The consequences of delinquency in terms of education are broad and include problems related to language and literacy (Willcutt & Pennington, 2000), teacher stress (Chaplain, 2003), and poor academic achievement (Calkins, Blandon, Williford, & Keane, 2007). Therefore, it is in the interests of schools to try to intervene to prevent and reduce youth offending.

### School influences on offending

Research on school‐level processes has primarily focused on educational outcomes. However, schools also play a pivotal socialization role: They are usually the first formal social environments that children experience. According to Hirschi's (1969) Social Bond theory, when bonds between children and key establishments or individuals in their lives (e.g., schools, family) become broken, this can increase the risk of delinquent behaviour. Similarly, Sampson and Laub's (2005) life course theory explains the importance of social institutions in adolescents lives, particularly, when a protective family environment is missing. Specifically as bonds weaken between establishments/key individuals and the adolescent, the adolescent is less likely to conduct themself in accordance with family or school rules and more likely to behave in ways that they feel are in their own personal interests. Conversely, when bond between school and adolescent is strong, this can exert a protective effect against offending behaviour; for example, the behaviour of principals, teachers, and other school staff contributes to reinforce rules and transmit social values. However, the bonding effect can sometimes exert detrimental effects, if staff instead behave in authoritarian ways that are contrary to internal school inclusivity and openness policies, thus communicating a different set of rules and values to the ones formally stated and inhibiting pupils' commitment and attachment to the school.

Consistent with these theories, both school and familial effects are related to developmental outcomes such as offending (e.g., Patterson, Reid, & Dishion, 1992; Rutter & Maughan, 2002 for a review). Evidence from the literature supports the notion that the school environment can affect young people's non‐educational outcomes. Research has particularly focused on the effects of school context on children and adolescents' development. For example, school commitment (Chan & Chiu, 2015; Monahan, VanDerhei, Bechtold, & Cauffman, 2014) and school safety (Lenzi *et al*., 2015) are associated with delinquency/gang involvement, and pupils' attachment to their school in adolescence has been linked to later patterns of offending (Savolainen *et al*., 2012). Liljeberg, Eklund, Fritz, and af Klinteberg (2011) reported that poor school attachment and commitment recorded at age 14 years predicted higher levels of delinquency for 16‐year‐old boys, although the same longitudinal relationships were not observed among girls. Other characteristics of the school environment can also influence developmental processes. For example, the balance of school intake across socio‐economic strata, ethnic composition of pupils' population and their diversity and school size have all been observed to influence developmental processes (Le & Stockdale, 2011; Lenzi *et al*., 2015).

While school‐level processes are generally considered not capable of eliminating entirely the effects of family experiences (Dufur, Hoffmann, Braudt, Parcel, & Spence, 2015; Duncan, Boisjoly, & Harris, 2001; Duncan & Murnane, 2011; Solon, Page, & Duncan, 2000), nevertheless, they can provide remedial processes or resources that may counter the influence of detrimental family processes on offending behaviour (Barnert *et al*., 2015; Hoffmann & Dufur, 2008). The socialization role of schools has become more of a formally recognized role in Northern Ireland since the personal, social, and health education curriculum was made statutory in all grant‐aided primary and post‐primary schools in 2007. The addition of non‐academic skills to the curriculum is consistent with the social development model developed by Hawkins (1985), who argued that the most important socialization units affect individuals sequentially – these being the family, schools, peers, and community influence.

### Evidence gaps

Despite the interest in school‐level effects, the literature on this topic highlights some limitations that are common to many studies. One key limitation in the extensive body of literature in the area demonstrates that most of the associations between school‐level processes and adolescents' behaviour are investigated cross‐sectionally and are therefore not able to provide much information on the direction of the causal influence of these processes. A more fundamental limitation shared by many studies that have investigated school effects is that many of these studies investigated school‐related processes (e.g., school attachment) at the individual level only (i.e., pupils within schools) and did not include school‐level measures in their models. In order to understand the way that contextual processes influence individual behaviour, it is necessary to use multilevel modelling to go beyond the individual and investigate processes at the interindividual level. Multilevel models separate the effects of observed and unobserved group characteristics and thus gauge the role that group behaviour and attitudes at the school‐level can exert on individual's behaviour. Such an approach is very much consistent with the findings of Manuel and Jorgensen's (2012) systematic review of 56 studies on youth offending which highlighted that the most effective interventions are those which use multiple delivery modes such as focusing on the broader environment (e.g., school environment) in addition to targeting the individuals themselves. For example, the overall values of school peers may be important in influencing the values and behaviour of young people particularly during adolescence, when young people become more independent from their family (Albert, Chein, & Steinberg, 2013; Larson, Richards, Moneta, Holmbeck, & Duckett, 1996).

### Current study

Using data from the Belfast Youth Development Study (BYDS) allowed the present research to investigate individual and interindividual processes. The BYDS study sampled the majority of pupils in 42 participating schools, thus allowing for the construction of school‐level variables reflecting shared attitudes, beliefs, and common conduct within school. The focus was on school contexts in year two of post‐primary education because it is reasonable to expect that by this time young people had enough time to form social bonds and develop patterns of behaviour in their new school settings. The focus was on school‐level processes in mainstream schools; while mainstream schools do have pupils with special educational needs, their principal role is to provide educational provision for those without special educational needs. There were too few participants attending alternative education providers (e.g., special schools) to examine variation by type of provision. Using a multilevel modelling approach, the study aimed to (1) estimate the importance of school‐level factors in explaining variability in offending trajectories in adolescence; (2) investigate individual and school‐level factors that may act as risk factors for offending and those that, conversely, may buffer against risk or play a protective role; and (3) investigate interactions between individual‐level characteristics and school‐related behaviour and school environment to test whether some school environments moderate the associations between individual characteristics and offending.

## Method

### Participants

**Figure 1:**
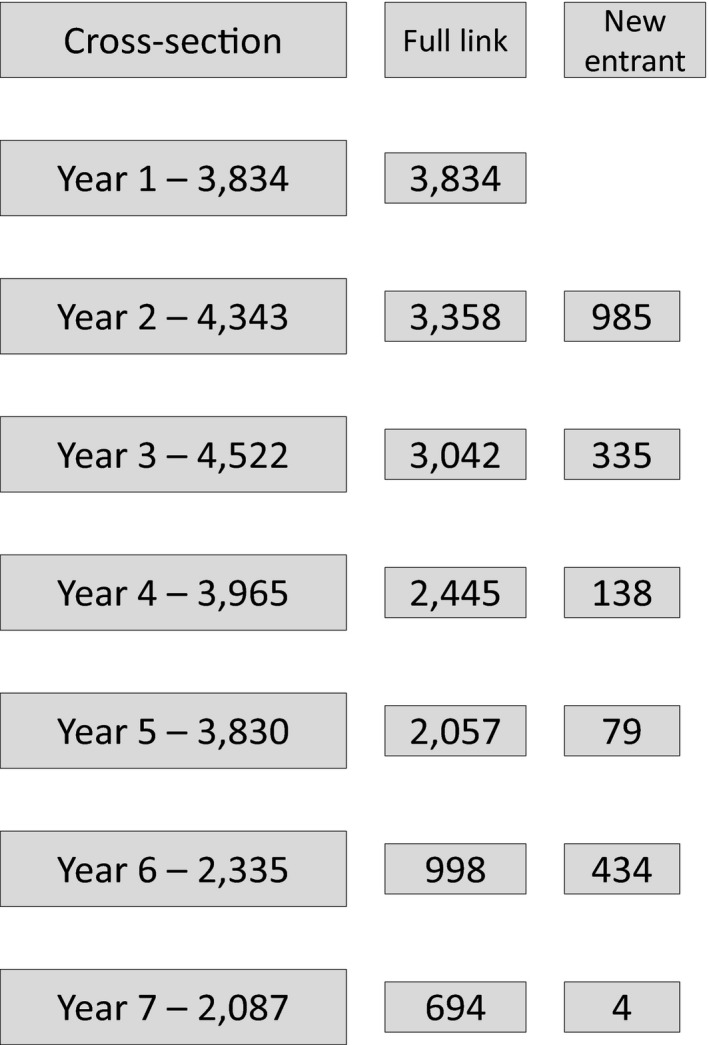
Number of participants by data collection wave.

The participants were young people attending mainstream post‐primary schools in Northern Ireland who took part in BYDS, which spanned seven waves (W) (see Higgins *et al.*, 2018 for a detailed description of the BYDS sample). The present study uses data from the first five waves which were separated by yearly intervals covering the period from W1 in 2001 (12 years old) to W5 in 2005 (16 years old). In total, 42 mainstream schools took part in the study. Letters were sent to all parents from participating schools explaining the study and giving them the option to remove their child from the study. The number of participants in every data collection sweep, as well as the number of new entrants in each wave, is reported in Figure 1. The main analysis in the present study (i.e., multilevel growth models) is based on the 4,049 who provided complete data on the model covariates.

### Measures

#### School behavioural variables

At age 13, a series of questions were asked in relation to school commitment, teacher–pupil relationships, and school problem behaviour. Pupils reported on the frequency of these behaviours on a 5‐point scale. Exploratory factor analyses were used to investigate the underlying structure of the items (see Appendix A for details). Individual student's scores on each latent construct were extracted from the model assuming three related underlying factors; some items were reversed so that higher scores corresponded to ‘positive’ behaviour (e.g., stronger attachment). Successively, school‐level characteristics were derived by calculating the means of the constructs within schools.

The three latent constructs uncovered in analysis were named: attachment; commitment; and aspirations; only attachment and commitment were retained for the present analysis (see Appendix A for details).

##### School attachment

This included 5‐point Likert items related to feelings of belonging at school (internal consistency = .78), with higher scores indicating greater levels of school attachment.

##### School commitment

These 5‐point Likert items tapped into the students' personal effort and investment in school (internal consistency = .71); higher scores indicated greater levels of school commitment.

#### School safety

A question relating to frequency of fights in the school grounds has been deemed to be an indicator of school safety and hence was considered on its own. Students reported whether or not they had been involved in fights within the school grounds in the last year or not.

#### School characteristic variables

Characteristics of schools measured included *school gender* (boys only, girls only, or co‐educational), *number of pupils, grammar status* (grammar or secondary school). Grammar schools require pupils to pass academic selection tests when 11 years old to gain entry. By contrast, grades are not used by secondary schools to select pupils.

#### Neighbourhoods and neighbourhood deprivation

At age 15, respondents were asked to indicate the postcode of their residence. Using available postcodes, the Central Postcode Directory was merged with the BYDS data to identify the Super Output Area (SOA) that each participant lived in. These SOAs were then used to merge the Northern Ireland Multiple Deprivation Measure (Northern Ireland Statistics & Research Agency, 2005) into the data, giving an SOA level deprivation score for each participant. The Northern Ireland Multiple Deprivation Measure (Northern Ireland Statistics and Research Agency, 2005) is a summary measure of seven types of area‐based deprivation including income; employment; health and disability; education, skills and training; proximity to services; living environment; and crime and disorder.

#### Individual and family covariates

##### Receipt of free school meals (FSM)

When 13 years old, participants were asked to indicate whether they received FSM (yes/no), and this was used as a proxy of socio‐economic deprivation.

##### Living arrangements

Respondents were also asked to indicate with whom they lived for most of the year (e.g., father and mother; mother and step‐father; mother only) at age 13 years. The resultant variable was categorized as follows: Respondent lived with both biological parents; lived in a ‘reconstituted family’ (one biological parent and a step‐parent, e.g., mother's partner); lived with a single biological parent (e.g., mother only); or lived in some other type of arrangement (e.g., foster parents; looked after by grandparents).

##### Parental monitoring

The parental monitoring subscale of the parental monitoring questionnaire (Kerr, Stattin, & Burk, 2010; Stattin & Kerr, 2000) was used at age 13 years to measure how much knowledge parents had regarding the young person's activities outside the home: Higher scores indicate better parental knowledge. The parental monitoring subscale included nine items scored on 5‐point Likert scales indicating the frequency of each behaviour (scale range 0–36). Some item scores were reversed to ensure higher scores indicated higher levels of monitoring, and total scores for each scale were derived by summing item scores. Stattin and Kerr (2000) have reported that the child report parental monitoring subscale has a good internal reliability (Cronbach's alpha, .86) and test–retest reliability (.83). In the present analysis, internal consistency of this scale was also good (.86). The parental monitoring score was used as a covariate in the analyses.

##### Inventory of parent and peer attachment (IPPA) parental sub‐scale

The parental subscale of the IPPA questionnaire (Armsden & Greenberg, 1987) was administered at W1, W3, and W4 (Cronbach's alpha .78–.95). This scale assessed young people's perceptions of the positive and negative affective/cognitive dimension of their relationship with their parents, and particularly how these figures served as sources of psychological security. Higher scores on the scale indicated worse attachment and worse relationships of young people with their parent(s). In W1, 12 items on 3‐point Likert scales were included in the scale (range 0–24), while the W3 and W4 scales comprised 28 items on 5‐point Likert scales (range 0–112). In the analyses, average scores across the three assessments were used as a covariate.

##### Offending behaviour

In each BYDS wave, respondents were asked to report the frequency of their involvement in 14 offending acts (see Appendix B, Table 10 for items). Good internal consistency was evident at all waves (Cronbach's alpha .81–.86) for the offending behaviour latent construct. In W1, respondents were asked whether they had ever been involved in any of these acts; from W2 onwards, respondents were asked about their recent (i.e., last 12 months) involvement in the offending behaviour types listed. Furthermore, whenever a respondent reported recent involvement in one offending act, they were also asked to report the frequency of this involvement in the last year on a Likert‐type scale (1–2; 3–5; 6–9; or 10 times or more); from this, a 5‐point Likert scale was constructed for each item. Offending factor scores were calculated by deriving a measurement invariance model for offending items between W2–5 (see Appendix B for details). The factor scores estimated from this measurement model were then used in further analyses (see multilevel growth models section).

Tables 1 and 2 present descriptive data for the measures included in the present research.

**Table 1:** Sample descriptive statistics for categorical data

	*N*	%
School type		
Grammar	1,884	47
Non‐grammar	2,165	53
School sex		
Boys only	1,079	27
Girls only	1,361	34
Co‐ed	1,609	40
FSM		
No	3,046	75
Yes	1,003	25
Living arrangements		
Biological parents	3,100	77
Reconstituted family	316	8
Single biological parent	581	14
Other	52	1
All participants	4,049	100

**Table 2:** Sample descriptive statistics for continuous data (raw scores)

	Mean	*SD*	*N*	Min	Max
Offending W3	5.64	7.02	3,716	0	56
Offending W4	4.72	6.59	3,193	0	56
Offending W5	4.55	6.57	3,101	0	56
Parental attachment W1	5.05	4.00	3,139	0	23
Parental attachment W3	35.02	20.61	3,623	0	111
Parental attachment W4	35.73	21.41	3,128	0	110
Parental monitoring W2	25.86	7.95	4,049	0	36
School attachment W2	14.96	5.98	3,838	0	28
School commitment W2	12.59	2.94	3,926	0	16

### Plan of analyses/analytical plan

The analyses proceeded in two steps. Firstly, as described above, factor scores were derived for offending behaviour using a measurement model (see Appendix C). We then considered the offending scores estimated at W3, 4, and 5 as the dependent variable.

Subsequently, we used multilevel growth models to test the association of intercept and rate of change of the offending outcome on school‐level and individual‐level predictors at W2. Because individual and contextual variables were assessed at a time that preceded the offending measures, we could argue that the direction of influence was from school processes to offending. The multilevel models were estimated using maximum‐likelihood estimation to handle missing data in the outcome (offending). Overall, these analyses included 4,049 participants for whom offending factor scores had been estimated in the measurement model and who had provided data for the predictors of interest. The models presented nested data with measurement occasions nested within individuals nested within schools. Linear change of outcomes with time was assumed: To this end, a square‐root‐transformation was applied to the time variable. The models also assumed normally distributed random effects at the school (level 3) and student level (level 2) to adjust for clustering at these levels. A formal description of the model is provided in Appendix D.

## Results

### Descriptive statistics

Table 3 reports standardized descriptive statistics for individual‐ and school‐level offending factor scores for W3–5. In comparison with W2 whereby the mean of offending was 0 by convention, offending increased in W3 and then decreased substantially in successive years. Table 4 shows school‐level mean offending scores by grammar status and school gender; conspicuous differences can be noticed between different types of schools.

**Table 3:** Descriptive statistics for individual‐ and school‐level offending factor scores by wave

	Individual level		School level	
W (age)	Mean	*SD*	Mean	*SD*
W3 (14 years)	.06	0.64	.08	0.20
W4 (15 years)	−.13	0.70	−.12	0.20
W5 (16 years)	−.26	0.73	−.24	0.21

**Table 4:** Average, school‐level offending scores by year and by school characteristics

	Wave (age)			
School characteristics	W2 (13 years)	W3 (14 years)	W4 (15 years)	W5 (16 years)
Grammar	−0.13	−0.03	−0.23	−0.35
Non‐grammar	0.06	0.15	−0.05	−0.19
Boys	0.24	0.27	0.09	0.02
Girls	−0.23	−0.10	−0.29	−0.44
Co‐ed	0.04	0.07	−0.15	−0.28

### Multilevel growth models

Multilevel models were run on the W3–5 offending factor scores as derived from the factor analysis (outcome variable). The factor scores were standardized within W3–5 so that the dependent variable represented differences from the group mean factor score between 14 and 16 years. The time variable used was data collection year: This was transformed (square root of time) and centred at W3: In this way, changes are relative to 14 years old. Singer and Willett (2003) suggest that transforming the level‐1 time variable is an optimal strategy for dealing with non‐linearity of individual change. This transformation was chosen based upon the shape of the data, using the ‘ladder of transformation’ as a guideline. The transformation used provided an alternative metric for age, which allowed individual change trajectories to be akin to linear ones. Analyses revealed that many of the covariates (e.g., gender, FSM) predicted missingness on the outcome variable indicating that it is likely that the MAR assumption was met. A summary of results of the five models tested is reported in Table 5.

**Table 5:** Results of multilevel growth models on general offending

	Model 1	Model 2	Model 3	Model 4	Model 5
*Fixed effects*					
Intercept	.02 (.04)	.26*** (.04)	.39*** (.08)	−.01 (.08)	.13 (.08)
School‐level school behaviours					
Sch attachment			−.07 (.04)	.01 (.03)	−.02 (.03)
Sch commitment			−.15* (.07)	−.11* (.06)	−.00 (.06)
Sch fights			.07 (.04)	.08* (.03)	.05 (.04)
Individual‐level gender (covariate)					
Female				Ref	Ref
Male				.40*** (.04)	.21***(.03)
Individual‐level living arrangements (covariate)					
Biological parents				Ref	Ref
Reconstituted family				.13** (.04)	.07 (.04)
Single biological parent				.04 (.03)	.01(.03)
Other				.04 (.10)	−.08(.09)
Individual‐level covariates					
FSM				.12 ***(.03)	.04(.03)
Parental attachment				.17*** (.01)	.15***(.01)
Parental monitoring				−.37*** (.01)	−.20***(.01)
Individual‐level school behaviours					
Sch attachment					−.03* (.01)
Sch commitment					−.33*** (.02)
Sch fights					.20*** (.03)
School gender					
Boys			Ref	Ref	Ref
Girls			−.12 (.11)	.19 (.10)	.06 (.11)
Co‐ed			−.15* (.07)	.03 (.06)	−.06 (.06)
Grammar status					
Grammar			Ref	Ref	Ref
Non‐grammar			−.02 (.08)	−.05 (.06)	−.03 (.07)
Grammar status × School‐lev commitment					
Grammar			Ref	Ref	Ref
Non‐grammar			.09 (.06)	.06 (.05)	.05 (.06)
FSM × Sch.Lev fights					
No FSM				Ref	Ref
FSM				−.10** (.03)	−.06* (.03)
Cross‐level interactions					
Sch‐lev commitment × Ind‐lev committment					−.02* (.01)
Cross‐classified					
Deprivation					
*Rate of change*					
Intercept	–	−.93*** (.03)	−.90*** (.08)	−1.06*** (.09)	−1.04*** (.09)
School‐level behaviour					
Sch.Committment			.15 (.07)	.13 (.07)	.14 (.07)
Individual‐level Beh					
Sch committment					−.04 (.02)
Individual‐level gender (covariate)					
Female				Ref	Ref
Male				.18** (.06)	.16**(.06)
Individual‐level covariate					
Parental monitoring				.05** (.02)	.07**(.02)
School gender					
Boys			Ref	Ref	Ref
Girls			−.19* (.08)	−.01 (.10)	−.03 (.10)
Co‐ed			−.17** (.06)	−.09 (.07)	−.10 (.07)
Grammar status					
Grammar			Ref	Ref	Ref
Non‐grammar			.07 (.10)	.06 (.09)	.06 (.09)
Grammar status × School‐Lev commitment					
Grammar			Ref	Ref	Ref
Non‐grammar			−.15 (.08)	−.14 (.08)	−.14 (.08)
*Variance components*					
School level					
Initial status	.07 (.02)	.07 (.02)	.01 (.00)	.01 (.00)	.01 (.00)
Rate of change		.02 (.007)	.01 (.01)	.01 (.01)	.01 (.01)
Covariance		—	—	—	—
Individual level					
Initial status	.76 (.02)	.71 (.02)	.71 (.02)	.47 (.01)	.38 (.01)
Rate of change		.75 (.03)	.75 (.03)	.74 (.03)	.74 (.03)
Covariance		.07 (.02)	.07 (.02)	.07 (.01)	.07 (.01)
Within person	.16 (.00)	.06 (.00)	.06 (.00)	.06 (.00)	.06 (.00)
SOA					
*R* ^2^					
*R* ^2^ sch‐lev initial status			.90	.30	−.65
*R* ^2^ sch‐lev rate change			.36	.05	.01
*R* ^2^ ind‐lev initial status			.00	.35	.18
*R* ^2^ ind‐lev rate change			.00	.01	.00
*R* ^2^ within person		.63	.00	.00	.00

Note

Model 1 = unconditional means model; Model 2 = unconditional growth model; Model 3 = school‐level predictors; Model 4 = conditional school‐level predictors; Model 5 = conditional individual‐level predictors.

*p < .05; **p < .01; ***p < .001.

#### Model 1

First a mean‐only model was used to assess how much variability in the outcome was explained by interindividual differences (level 2) and how much variability was explained by differences between schools (level 3). The ICC indicated an intracluster correlation equal to 0.07 at school level and an ICC equal to 0.84 at the student level. A likelihood ratio test revealed that including school variation in the model resulted in a significant increase of fit, LRT χ^2^(1) = 253, *p *<* *.001, compared to the 2‐level wave and pupil model.

#### Model 2

The unconditional growth model enabled the general direction and growth rate in offending scores to be measured, by adding time to the model. Time was associated with a significant decrease in offending scores across time: Each time unit was associated with a reduction of – 0.93 *SD* units in offending scores. The inclusion of time explained a considerable amount of variance in offending within individuals (63%).

#### Model 3

Successively, all W2 school‐level behavioural and characteristic variables considered were entered as predictors of W3 score (initial status) and of growth rate. This model explained approximately 90% and 36% of variation across schools in initial status and growth rate, respectively. The commitment school behavioural variable was associated with W3 initial scores. A 1 *SD* increase in school‐level commitment was associated with a 0.15 *SD* reduction in W3 offending scores. School gender was also associated with W3 scores and growth rate. Compared to boys‐only schools, co‐ed schools displayed lower offending in W3, and co‐ed and girls‐only schools had a steeper offending slope compared to boys‐only schools.

#### Model 4

Student‐level covariates were then added to estimate the school‐level effects on the outcome trajectory while controlling for other characteristics; this led to a significant improvement in model fit. The inclusion of the student‐level variables in the model explained 35% and 1% of between‐student variation in initial offending score and growth rates, respectively. A further 30% between‐school variation in initial scores was also explained by the inclusion of the interaction between FSM eligibility and school fights proportion.

There was a significant association between gender and W3 scores as well as between gender and offending growth rate: Male students reported higher scores in W3 compared to females, and they also displayed a shallower decrease in offending compared to females. There was also a significant association between FSM eligibility and initial offending scores, with students entitled to FSM displaying higher scores. There was a significant association between initial W3 offending scores and living arrangements (students in reconstituted families displayed higher initial scores compared to students living with both biological parents), students' family attachment (worse attachment was associated with higher offending scores), and parental monitoring (poorer parental monitoring being associated with higher initial offending scores). Finally, there was an association between parental monitoring and offending growth rate, with students reporting better parental monitoring displaying a less steep decrease in offending between W3–5.

**Figure 2:**
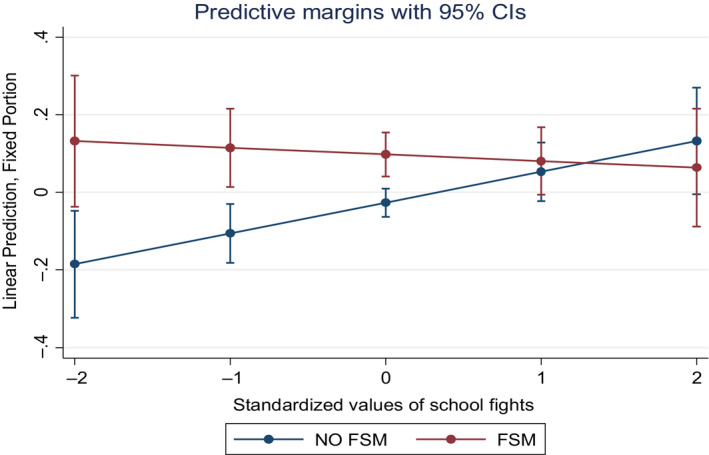
Predictive margins (and 95% CI) of general offending initial level by FSM eligibility and school fights. [Colour figure can be viewed at wileyonlinelibrary.com]

After controlling for student‐level covariates, there was a significant interaction between school fights at the school‐level and FSM eligibility. This interaction illustrated in Figure 2 highlights that for students not exposed to higher levels of economic deprivation (non‐FSM eligible), a lower proportion of school fights at the school‐level was associated with lower offending.

After including individual‐level predictors in Model 4, the association between school‐level fights and initial offending was significant (1 *SD* increase in school‐level fights proportion being associated with .08 *SD* increase in offending scores).

#### Model 5

In the final model, student‐level school‐related behaviour was controlled for. All three school‐related behaviour categories were significantly associated with offending initial scores in the study: A 1 *SD* increase of student attachment was associated with a 0.03 reduction in initial offending scores (a significant, but not particularly strong association), while a 1 *SD* increase in individual school commitment was associated with a 0.33 *SD* reduction in initial offending scores. Reporting being involved in school fights was associated with a 0.20 *SD* increase in offending initial score in the study. Lower individual‐level commitment was associated with higher initial levels of offending at age 14 if the school‐level ethos was of higher commitment.

Inclusion of student‐level school behaviour variables attenuated the effects of school‐level variables, which, in Model 5, displayed no association with offending behaviour. However, a 2‐way interaction between FSM eligibility and proportion of fights at school‐level remained significant.

Finally, further analysis using a cross‐classified approach on those participants that provided information on their residence (*N* = 3,338) was used to examine the proportion of variability attributable to schools and neighbourhoods.

ICC indicated that approximately 7% (LRT χ^2^(1) = 155, *p* < .001) of offending variability was explained by school differences, while only 2% (LRT χ^2^(1) = 8.32, *p* < .01) of offending variability was related to geographical differences.

## Discussion

### School and individual‐level predictors

The present investigation examined school influence on offending behaviour during mid‐adolescence (14–16 years). In total, school‐level variables explained 7% of offending behaviour variation. The results highlighted school‐level contextual processes and school characteristics which may explain school variation in offending. After controlling for student characteristics, only school fights were related to changes in offending behaviour, albeit via a complex relationship. Lack of safety at the school‐level may be particularly detrimental for young people not exposed to socio‐economic deprivation. While exposure to different levels of unsafe school environments seemed not to be associated with different levels of offending for young people exposed to socio‐economic deprivation, for those not exposed to deprivation, lack of safety was associated with higher risk of offending. In terms of individual‐level predictors, pupils who had high levels of commitment and attachment to their school and were involved in few fights reported lower levels of offending at 14 years old. Further, lower individual‐level commitment had a greater association with higher initial levels of offending at age 14 if the school‐level ethos was of higher commitment.

### Cost‐effectiveness implications

A large proportion of variation in youth offending behaviour was attributed to student‐level characteristics (83%). Hence while the present multilevel modelling results suggest that both individual‐level and school‐based interventions can play a role in tackling youth offending, where resources are limited an individual‐level approach is likely to be more cost‐effective than a school‐level approach. Using an individual‐level approach, children who are at risk of future offending would be specifically targeted with the aim of improving their school commitment, school attachment, and to prevent them from being involved in fights. While targeted individual‐level interventions in adolescence are rare, interventions to promote a caring and positive school ethos are more common in early education. For example, some primary schools in the United Kingdom put children at risk of poor social and developmental outcomes into nurture groups. As these children often have poor attachment to their family, the teacher and school try to address these attachment needs by building a secure and stable relationship with the child and promoting the image of the school as a safe environment (Boxall & Lucas, 2011). Directing efforts on these individuals in early life is likely to reduce youth offending as well as help these individuals avoid other adverse outcomes in physical and mental health domains and reduce the social and financial burdens taken on society by the most chronic offenders (Cohen & Piquero, 2009). The development of similar but age‐appropriate approaches in post‐primary education could therefore potentially help boost school commitment and attachment and reduce youth offending. Where additional resources are available, a school‐level approach could lead to additional, albeit, smaller reductions in offending in some schools. Indeed, pupils from schools with a nurturing and inclusive school ethos have been found to have better psychological well‐being development than pupils from schools not adopting such an approach but who have pupils with similar needs (e.g., Reynolds, MacKay, & Kearney, 2009). The present research shows that it may make sense to target resources for school‐level interventions at particular school types and these are not necessarily those that are currently prioritized by educational authorities. Specifically, the present results indicate that a wider roll‐out of intervention at school‐level would lead to greater benefits in certain types of schools (i.e., non‐deprived areas); in these schools, at‐risk children would also benefit from being surrounded by peers who do not engage in fights frequently. Indeed, many programmes to reduce offending are prioritized in deprived areas, such as the extended schools programme (Department of Education Northern Ireland, 2014); however, the present results suggest that greater gains could be obtained from paying relatively greater attention to less deprived schools. This unexpected finding particularly underscores the importance of gathering data and evidence which highlight some of the protective factors that would best reduce the likelihood of future violence.

### Limitations and future directions

The current study should be interpreted in the light of its methodological considerations and limitations. The main limitation is that the offending data were gathered through self‐report measures and are thus subject to all the known shortcomings surrounding such methodology including social desirability. It is possible that pupils were concerned about reporting offences for fear of being reported, or they may have confessed to acts they had not committed to portray a certain self‐image. It would not be possible to verify the reported offending data as most of the reported acts were minor and would have been unlikely to have resulted in police intervention (e.g., not paid the correct fare on a bus or a train). Nevertheless, rigorous steps were taken to minimize false reporting by emphasizing the confidentiality and anonymity of the research and the importance of the findings. A clear association between school‐related variables and youth offending has been identified and the longitudinal nature of the study suggests a causal relationship, although stronger evidence is needed to be more confident about the direction of the relationship. For example, on the basis of their longitudinal findings, Hoffmann, Erickson, and Spence (2013) have suggested that delinquent behaviour leads to poor attachment as opposed to the other way around. Future research using randomized control methodology to evaluate post‐primary interventions designed to promote a more positive school environment is needed so that a causal link between school context and future offending can be further validated. In addition, future research would benefit from implementing a long‐term follow‐up period into the design to assess the impact of the intervention on future offending.

### Conclusions

In summary, at present there is a lack of targeted individual‐level evidence‐based interventions designed to improve school attachment and commitment among adolescents at risk of offending. Targeted individual and family level interventions are likely to lead to the greatest reductions in delinquency and hence constitute a more cost‐effective approach than school‐level interventions. Where additional resources are available, in some school types, additional, albeit modest, reductions in offending may be possible through school‐level intervention designed to improve the overall level of pupil sense of safety within their schools. Issues associated with area‐level deprivation may be best addressed at a national or local level rather than a school level.

## Conflicts of interest

The authors declare that they have no conflict of interest.

## Appendix A

We ran the exploratory factor analysis of the school behavioural variables using Mplus 7.1 (Muthén & Muthén, 2012). A ‘sandwich estimator’ was used to adjust standard errors for clustering of students within schools. The resulting eigenvalues and the scree plot suggested a 4‐factor solution (Figure 3). This conclusion was also supported by a parallel analysis (Table 7). However, the minimum average partial correlation analysis suggested that two components be retained. While the fit indices of the one‐ and two‐factor solutions reported in Table 6 indicate inadequate fit of the models, the three‐factor solution displayed adequate fit based on commonly used indices. Comparing the three‐ and four‐factor solution also indicated that the fourth factor in the latter solution had only two items that displayed substantial loadings on it. Thus, taking into account the fit indices of the different solutions and the need for parsimony, we decided to consider a three‐factor solution in further analyses (Tables 8 and 9).

**Figure 3.** Scree plot. [Colour figure can be viewed at wileyonlinelibrary.com]

**Table:** **Table 6.** Eigenvalues for sample correlation matrix and fit statistics

	Eigenvalues	Chi‐square (*p*)	RMSEA	CFI	TLI
1 Factor	6.346	<.001	0.11	0.85	0.83
2 Factors	2.095	<.001	0.08	0.93	0.91
3 Factors	1.536	<.001	0.07	0.96	0.93
4 Factors	1.087	<.001	0.04	0.99	0.98
5 Factors	0.762	<.001	0.03	0.99	0.99
6 Factors	0.686	<.001	0.03	1.00	0.99

Note

CFI = Comparative Fit Index; RMSEA = Root‐mean‐square error of approximation; TLI = Tucker–Lewis Index.

**Table:** **Table 7.** Factor analysis, parallel analysis, and minimum average partial correlations

	Factor analysis eigenvalues	Eigenvalues averaged over 50 replications	Minimum average partial correlations
1	5.377	5.259	0.038
2	1.974	1.878	0.021
3	1.440	1.362	0.022
4	1.096	1.036	0.025
5	0.805	0.758	0.037
6	0.740	0.705	0.050
7	0.646	0.624	0.072
8	0.608	0.600	0.092
9	0.555	0.560	0.122
10	0.523	0.539	0.142
11	0.469	0.497	0.215
12	0.465	0.505	0.343
13	0.430	0.483	0.401
14	0.361	0.430	0.660
15	0.334	0.418	1.000
16	0.176	0.280	

Key statistics for the three‐factor solution were as follows: RMSEA = 0.067; CFI = 0.96; TLI = 0.93; SRMR = 0.041. The estimator used in this analysis was WLSMV which uses a pairwise approach to handling missing data. All factors had good internal consistency and manifest variables loaded significantly onto their respective factors and factors correlated positively and significantly with each other.

These three factors derived from the factor analysis of the school variables loaded substantially with the following items:

Attachment: I think going to school is a waste of time; I never take school seriously; I am fed up with school; I don't like the subjects I do; I like school; I am always willing to help the teacher; I like my teachers;
Commitment: I am quiet in class and get on with my work; since the start of the year has skipped or bunked of class; since the start of the school year has been in trouble with the principal; since the start of the school year has been in detention;
Aspirations: I want to leave school at 16; I want to do GCSEs; I want to do GNVQs; I want to do ‘A’ levels; I want to go to university after school.

Only the attachment and commitment factors were retained for a number of reasons. Firstly, commitment and attachment were better‐defined constructs (the items reflected definitions used in previous research). Furthermore, these constructs that had been previously identified in the literature as playing an important role. So, in this case, the rationale was both theoretical (e.g., aligns with Hirschi's, 1969 social bond theory) and backed up by good qualities of these measures (e.g., their internal consistency, alpha, reported in the Methods). The other construct (Aspirations) was less well defined both in theoretical terms and in statistical terms, as this included items that had some dependency (e.g., ‘A’ levels are a pre‐requisite for university entry).

## Appendix B

**Table:** **Table 8.** School behaviour exploratory factor analysis loadings

Item	Factor 1	Factor 2	Factor 3
School is a waste of time	0.639*	0.171*	−0.013
Never take school seriously	0.422*	0.138*	0.048
Fed up with school	0.657*	0.101*	−0.128*
Don't like the subjects	0.473*	0.043	−0.070*
Like school	0.697*	0.131*	0.024
Quiet in class and get on with my work	0.226*	−0.019	0.474*
Skipped or bunked off class	0.091*	−0.001	0.629*
Been in trouble with the principal	−0.112*	0.028	0.857*
Been in detention	0.017	0.037	0.735*
Willing to help the teacher	0.597*	−0.019	0.197*
Like my teachers	0.570*	−0.017	0.203*
Want to leave school at 16	0.164*	0.674*	0.015
Want to do GCSEs	0.029	0.919*	−0.029
Want to do GNVQs	−0.032*	0.904*	−0.062*
Want to do A levels	−0.012	0.975*	0.026
Want to go to university after school	0.013	0.817*	0.083*

Note

Factor 1 = attachment; factor 2 = aspirations; factor 3 = commitment.

**p* < .05.

**Table:** **Table 9.** Correlations between factors

	Factor 1	Factor 2	Factor 3
Factor 1	1.000	–	–
Factor 2	0.359*	1.000	–
Factor 3	0.456*	0.442*	1.000

Note

Factor 1 = attachment; factor 2 = aspirations; factor 3 = commitment.

**p* < .05.

## Appendix C Offending factor analysis

The factor analysis of the offending items suggested that items in each year loaded onto a single dimension. We therefore constructed a measurement model to investigate changes in an underlying latent construct. Namely, we considered that adolescents' self‐report of involvement in the different offending acts considered were manifest variables of an underlying construct, which had a causal effect on the manifest variables. This reflective indicator model was informed by theories that conceptualize offending as a dimension with strong links with temperamental propensities, and a result of developmental processes that may affect the adolescent's ability to control emotion and behaviour (Moffitt, 1990; Rutter, 2003). Namely, offending may be the result of different biological and psychological processes that unfold over time and that provide a propensity for engaging in offending behaviour. Of course, there is debate over the nature of the causal processes behind offending behaviour, and therefore, it must be acknowledged that the approach used here may fit less well with other theories such as the evolutionary perspective which views aggression as a form of adaptation (e.g., Bjorklund & Hawley, 2014).

The measurement model was estimated using weighted least square with parameter estimates with standard errors and a mean‐ and variance‐adjusted chi‐square test statistic that used a full weight matrix (WLSMV). This estimator is considered more appropriate to analyse categorical variables that display asymmetric distributions. A theta parameterization was used to obtain residual variances in the model (Muthén & Muthén, 2012). The drawback of using a WLSMV estimator lies in the fact that missing data are handled in a less efficient way compared to ML methods: Weighted least‐square approaches require a pairwise present approach (Muthén, Muthén, & Asparouhov, 2015). However, the model constructed allowed estimation of parameters and scores for the vast majority of the participants in the study (5,221 out of 5,371), and therefore, we considered that the extent of missing scores was offset by providing enough information that allowed the estimation to be carried out for most of the participants.

Covariance between similar items across years was allowed in the model. Different models were tested and compared using model‐fit statistics (e.g., CFI) as well as chi‐square difference tests (see Appendix C).

The models tested more stringent assumptions (e.g., metric, scalar, and residual invariance) in turn. These suggested a better fit was provided by a scalar model whereby the indicators were invariant across age (ages 12–16), thresholds of categorical items were also invariant across age, but residual variance of indicators changed across age. The final model and its constraints are represented schematically in Appendix C. Notably, the latent variables are scaled to an observed variable (the first variable in the list, fare dodging), while the factor loadings of the manifest variables on the latent variables were constrained to be equal across measurement occasions; this assists interpretability, as the latent variable has the same metric as the scaling indicator (Bollen, 1989) Finally, the underlying factor mean at the first measurement point (age 12) was fixed at 0 by convention, while the mean of factors at successive measurement occasions was freely estimated. The model provided an indication of changes in offending behaviour. Statistics provided by Mplus version 7 indicated a good fit of this model: RMSEA = 0.009. CFI = 0.98; TLI = 0.99; WRMR = 1.536). Although the chi‐square test of model fit was significant, χ^2^(1,557) = 2,261.9, *p *<* *.0001, this is most likely due to the excess statistical power provided in the present research which used a large sample (Bollen & Curran, 2006). In data analyses, we used the factor scores estimated by this model as the dependent variables in the multilevel regression model. The purpose of using factor scores was to derive variables that took into account measurement error, and provide meaningful estimation of scores for those who left one or more offending items blank. Furthermore, in contrast to a sum of raw items score which treats all offending items equally, factor scores ‘weight’ offending acts. Specifically, using factor scores prevents incidents such as someone who reports dodging bus fares every day from being considered a worse offender than someone who commits a burglary but does not report any other offence. In contrast, items with larger loadings are provided a greater weight in generating the factor scores. The offending items were as follows:

**Table:** **Table 10.** Offending items

Offending act	Literal question. In the last 12 month, have you…
Fare Dodging	Not paid the correct fare on a bus or a train?
Shoplifting	Taken something from a shop or a store without paying for it?
Bad Behaviour	Behaved badly in a public place so that people complained or you got into trouble?
Joyriding	Stolen ridden in/on a stolen car or a van or on a stolen motorbike?
Steal from school	Taken money or something else that did not belong to you from school?
Weapon carrying	Carried a knife or weapon with you for protection or in case it was needed in a fight?
Damage	Deliberately damaged or destroyed property that did not belong to you (for example, windows, cars or streetlights)?
Burglary	Broken into a house or building to steal something?
Graffiti	Written things or sprayed paint on property that did not belong to you (for example, a phone box, car, building or bus shelter)?
Use of force	Used force, threats or a weapon to get money or something else from somebody?
Steal from home	Taken money or something else that did not belong to you from your home without permission?
Arson	Deliberately set fire or tried to set fire to someone's property or a building (for example, a school)?
Hitting others	Hit, kicked or punched someone on purpose to hurt of injure them?
Stealing from cars	Broken into a car of van to steal something out of it?

## Appendix D

**Table:** **Table 11.** Model fit for configural, metric, and scalar invariance models

Model	χ^2^ test of model fit	RMSEA	CFI	TLI	χ^2^ difference test
Configural	χ^2^(1,478) = 3,975, *p* < .001	0.02	0.95	0.95	–
Metric and scalar	χ^2^(1,640) = 4,079, *p* < .001	0.02	0.95	0.95	χ^2^ (162) = 214, *p* = .004

Note

The configural invariance model is the baseline model; the metric and scalar model is as per the configural model except the factor loadings and the item intercepts are constrained to be equal across time (note metric and scalar invariance were tested in one step as is customary when using the WLMSV estimator).

**Figure 4.** Offending behaviour measurement model (unstandardized). [Colour figure can be viewed at wileyonlinelibrary.com]

## Appendix E Formal description of multilevel growth models

The MLM included the following: a random intercept at pupil level, to model between‐pupil differences in offending at the first time point; a random slope for the wave coefficient, to model between‐pupil differences in rate of change of offending; and an unstructured covariance between the two, estimating the intercept–slope covariance at the pupil level. We also included a random intercept at the school level, to model between‐school offending differences; and a random slope with time at the school level, to model between school differences in rate of change in offending, fixing the covariance at zero.

*i = M*easurement Occasion; *j = P*upil; *k = S*chool

Offending_*ijk*_ = β_0_ + β_1_Year^Sqrt^ + β_1_[Individual‐Level Predictors]_*j*_ + β_2_[School‐Level Predictors]_*k*_ + *v*_0*k*_ + *v*_1*k*_Year^Sqrt^ + *u*_0*jk*_ + *u*_1*jk*_Year^Sqrt^ + e_*ijk*_

$$\left(\begin{array}{c} v0k\\ v1k \end{array}\right)∼N\left(0,\Omega v\right)\;\text{where}\;\Omega v=\left(\begin{array}{c} \sigma_{v0}^{2}\\ \sigma_{v1}^{2} \end{array}\right)$$

$$\left(\begin{array}{c} u0jk\\ u1jk \end{array}\right)∼N\left(0,\Omega u\right)\;\text{where}\;\Omega u=\left(\begin{array}{c} \sigma_{u0}^{2}\\ \sigma_{u01}\sigma_{u1}^{2} \end{array}\right)$$

$\sigma_{v0}^{2}$ = School – level residual variance in the intercept; $\sigma_{v1}^{2}$ = School – level residual variance in the slope; $\sigma_{u0}^{2}$ = Individual – level residual variance in the intercept; $\sigma_{u1}^{2}$ = Individual – level residual variance in the slope; σ_*u*01_ = Individual – level covariance between intercept and slope across all individuals in a school.
